# Deep learning tools for the cancer clinic: an open-source framework with head and neck contour validation

**DOI:** 10.1186/s13014-022-01982-y

**Published:** 2022-02-08

**Authors:** John C. Asbach, Anurag K. Singh, L. Shawn Matott, Anh H. Le

**Affiliations:** 1 Roswell Park Comprehensive Cancer Center, Elm and Carlton Streets, Buffalo, NY 14203 USA; 2 Jacobs School of Medicine and Biomedical Sciences, State University of New York at Buffalo, 955 Main Street, Buffalo, NY 14203 USA

**Keywords:** Deep learning, Software, Contouring, Head and neck, Open-source

## Abstract

**Background:**

With the rapid growth of deep learning research for medical applications comes the need for clinical personnel to be comfortable and familiar with these techniques. Taking a proven approach, we developed a straightforward open-source framework for producing automatic contours for head and neck planning computed tomography studies using a convolutional neural network (CNN).

**Methods:**

Anonymized studies of 229 patients treated at our clinic for head and neck cancer from 2014 to 2018 were used to train and validate the network. We trained a separate CNN iteration for each of 11 common organs at risk, and then used data from 19 patients previously set aside as test cases for evaluation. We used a commercial atlas-based automatic contouring tool as a comparative benchmark on these test cases to ensure acceptable CNN performance. For the CNN contours and the atlas-based contours, performance was measured using three quantitative metrics and physician reviews using survey and quantifiable correction time for each contour.

**Results:**

The CNN achieved statistically better scores than the atlas-based workflow on the quantitative metrics for 7 of the 11 organs at risk. In the physician review, the CNN contours were more likely to need minor corrections but less likely to need substantial corrections, and the cumulative correction time required was less than for the atlas-based contours for all but two test cases.

**Conclusions:**

With this validation, we packaged the code framework and trained CNN parameters and a no-code, browser-based interface to facilitate reproducibility and expansion of the work. All scripts and files are available in a public GitHub repository and are ready for immediate use under the MIT license. Our work introduces a deep learning tool for automatic contouring that is easy for novice personnel to use.

**Supplementary Information:**

The online version contains supplementary material available at 10.1186/s13014-022-01982-y.

## Background

Research interest in automatic organ segmentation for cancer treatment planning has rapidly increased in recent years. Manual contouring is a notably time-intensive process: it can take a trained physician several hours to fully contour a patient study [1]. Automatic methods aim to reduce this time, increase contour consistency, and improve contouring accuracy [2]. The increase in the number of studies on automatic contouring is attributable to advancements in the application of artificial neural networks, known as deep learning. For example, studies have demonstrated the accuracy and viability of the segmentation approach for lung cancer [3], prostate cancer [4, 5], head and neck cancer [6–9], and more [10, 11]. Thus, deep learning should no longer be viewed as an experimental technique in medical image processing. Nevertheless, the rapid expansion of the technique has divided researchers and clinicians.

New papers compete to prove state-of-the-art accuracy scores with new neural network algorithms, but the methods are often complex or unclear, the results can lack context, and the benefit to the average clinician is marginal. van Dijk et al. published a seminal study on deep learning segmentation for head and neck cancer in 2020 in which they performed thorough analyses and provided the results in a useful context; however, their method involves proprietary commercial software and the neural network structure was not clearly described [6]. Similarly, Liu et al. had promising results using a patch-based approach to contour the prostate region but did not detail the architecture, making it difficult to replicate their findings [5]. Zhong et al. described the architecture of their neural network in their report, but it is up to the reader to reproduce the code [12]. By contrast, van Rooij et al. provide the URL for the source code of the neural network they used, although crucial information about data pre-processing was described only in general terms [7]. Aside from these examples, other studies focus on evaluating the artificial neural network, treating it as a “black box” [13–15].

We set out to create a clear, reproducible deep learning framework that clinics can use to explore modern automatic segmentation. In addition to diagramming the neural network architecture, this requires data handling steps both before and after the neural network itself. Furthermore, the transformation to and from DICOM-compliant files is essential but often overlooked. To truly enable immediate reproduction and expansion of work in this area, the entire end-to-end code framework should be provided. This framework must include coded tools for data pre-processing, output cleaning, DICOM handling, and more.

Our research presents a complete convolutional neural network (CNN) workflow for automatic segmentation of 11 common organs at risk (OARs) in patients with head and neck cancer that uses only open-source Python tools freely available online. To validate the performance of the CNN, we benchmarked the results to those of a commercial atlas-based workflow that is available in our clinic, scoring each method quantitatively and qualitatively using accuracy metrics and expert physician review. To address the perceived shortage of ready-to-use deep learning tools, we made all necessary files available for this work, including the Python scripts which govern the data handling, CNN training, and contour generation processes, the pre-trained weights for immediate use, and the tools and templates to configure a simple web application to allow DICOM-compatible, browser-based utilization of the neural network.

## Methods

### Framework usability criteria

**Fig. 1:**
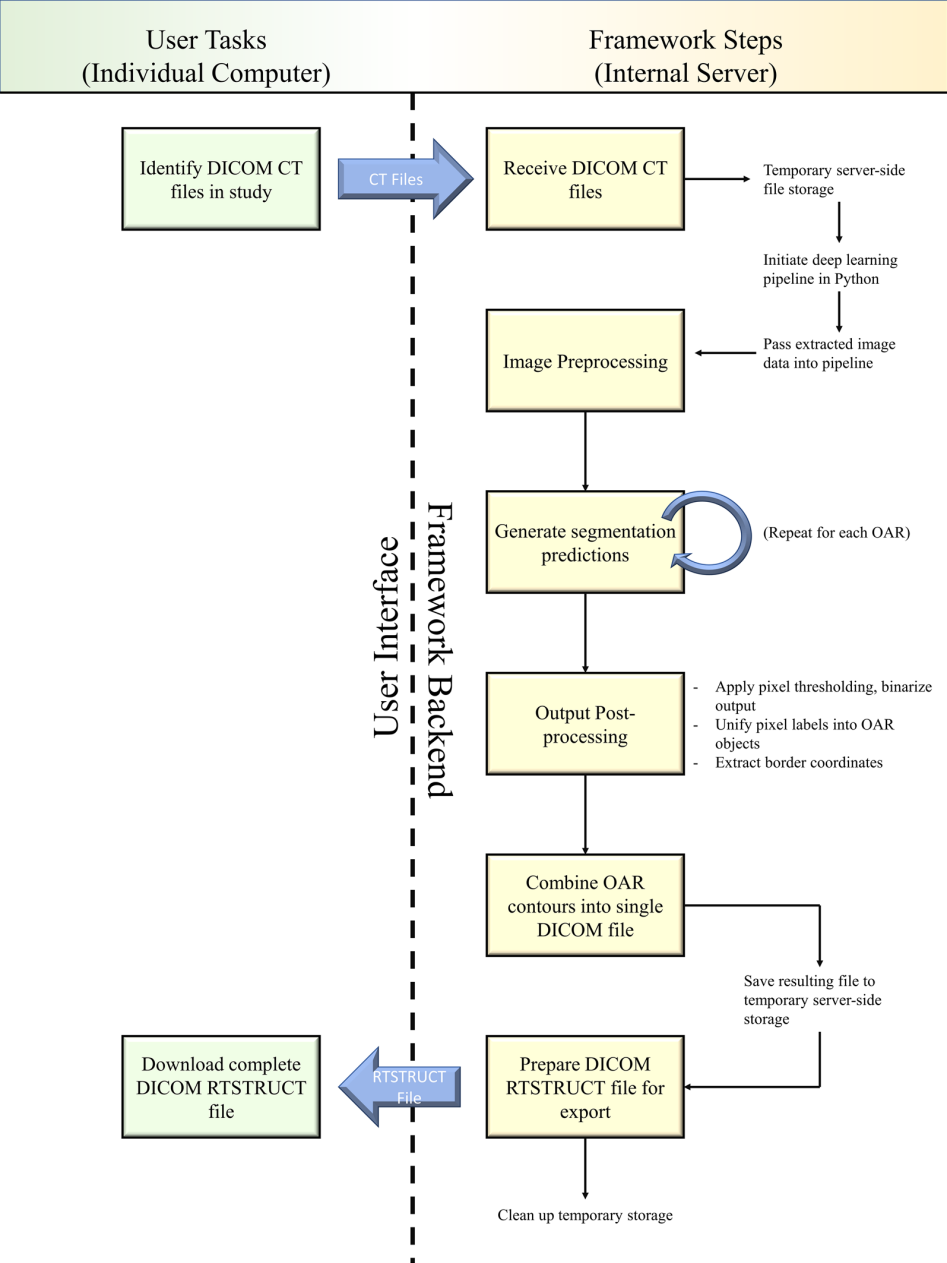
Schematic of data flow for deep learning framework operation

Several key usability criteria were established for an accessible deep learning contouring framework. First, the framework must be built to receive as input DICOM CT image files and produce as output a DICOM-compliant structure set file. This way, a user is not required to oversee any data preparation or manipulation but only needs to provide the CT files to the framework; the output is then ready to be reviewed and incorporated back into the treatment planning workflow. Second, the framework must support both contour creation and CNN retraining on new data. This makes replication very straightforward and it facilitates the exploration of other segmentation tasks using this approach. Finally, the framework must be built with tools to establish a browser-based interface for users without Python familiarity. This can be accomplished by configuring the framework on an internal server, with the files supporting the browser interface available with the core code of the framework. Figure 1 shows the design workflow for the framework as it should function when installed on an internal server managed by the clinic; note that there are only two configuration or decision points required of the user. The complete code for the deep learning pipeline as well as the code and design for the prototype browser-based interface can be found in a public GitHub repository [16].

### Data collection

The initial development and validation of the CNN used head and neck data. The retrospective planning CT images and accompanying OAR labels from 229 patients with head and neck cancer treated at our clinic between 2014 and 2018 were anonymized. Each study was acquired by helical scan with a large-bore GE Discovery CT simulator (GE Healthcare, Chicago, IL) using a head and neck protocol of 120 kVp, auto mA, large field of view, 2.5-mm axial slice thickness, and 512 × 512 pixel resolution. The manually defined OAR labels, approved by the physician at treatment planning, were used as the “ground truth” for CNN training. The 11 OARs examined—brain, brainstem, both cochleae, both parotid glands, both submandibular glands, larynx, brachial plexus, and spinal cord—were chosen on the basis of commonality in the dataset. After setting aside 19 patients with approved contours for all 11 OARs as unified test cases, each OAR had a training set from 140 to 210 patients. Table 1 shows the characteristics of patients in the training and test cohorts.

**Table 1:** Patient characteristics for training and test cohorts

Characteristic	Dataset, No. (%)	
	Training (210)	Test (19)
Age at diagnosis, median (range)	61 (40–84)	65 (53–88)
**Sex**		
Female	38 (18.1)	5 (26.3)
Male	172 (81.9)	14 (73.7)
**Primary tumor site**		
Base of tongue	45 (21.4)	2 (10.5)
Hypopharynx	10 (4.8)	1 (5.3)
Larynx	34 (16.2)	0 (0.0)
Nasopharynx	10 (4.8)	5 (26.3)
Oropharynx	21 (10.0)	4 (21.1)
Supraglottis	6 (2.9)	1 (5.3)
Tonsil	45 (21.4)	5 (26.3)
Other/unknown	39 (18.6)	1 (5.3)
**Tumor stage**		
TX	6 (2.9)	0 (0.0)
T0	12 (5.7)	1 (5.3)
T1	34 (16.2)	3 (15.8)
T2	50 (23.8)	5 (26.3)
T3	87 (41.4)	9 (47.4)
T4	10 (4.8)	1 (5.3)
Unavailable	11 (5.2)	0 (0.0)
**Node stage**		
N0	46 (21.9)	3 (15.8)
N1	37 (17.6)	2 (10.5)
N2	99 (47.1)	13 (68.4)
N3	17 (8.1)	1 (5.3)
Unavailable	11 (5.2)	0 (0.0)

Tumor staging ranges from TX/T0 (cannot be measured/found) to T4, with larger numbers indicating larger tumors. Node staging ranges from T0 (no cancer in nearby lymph nodes) to N3, with larger numbers representing greater presence of cancer in lymph nodes

All image pixels were converted to Hounsfield units using DICOM metadata and resampled to a pixel size of 1 mm^2^ to standardize the representation. Each image was cropped to the centermost 256 × 256 pixels and passed through a window/level (W/L) filter: a tissue filter (W/L: 400/40) for all OARs except spinal cord and brachial plexus, which were passed through a bone filter (W/L: 2,000/400). Finally, for each OAR’s training dataset, of the two-dimensional slices with no OAR data, every 20^th^ slice was included. This step is important for small OARs: a full CT study often exceeds 200 slices, but an OAR like the parotid gland might only be present on 10 slices. Taking only every 20^th^ slice that does not contain OAR data helps balance the distribution. This enables the CNN to learn a representation of images that do not contain any OAR data but does not skew the primary training focus of accurately labeling images that do.

### Neural network structure and training

**Fig. 2:**
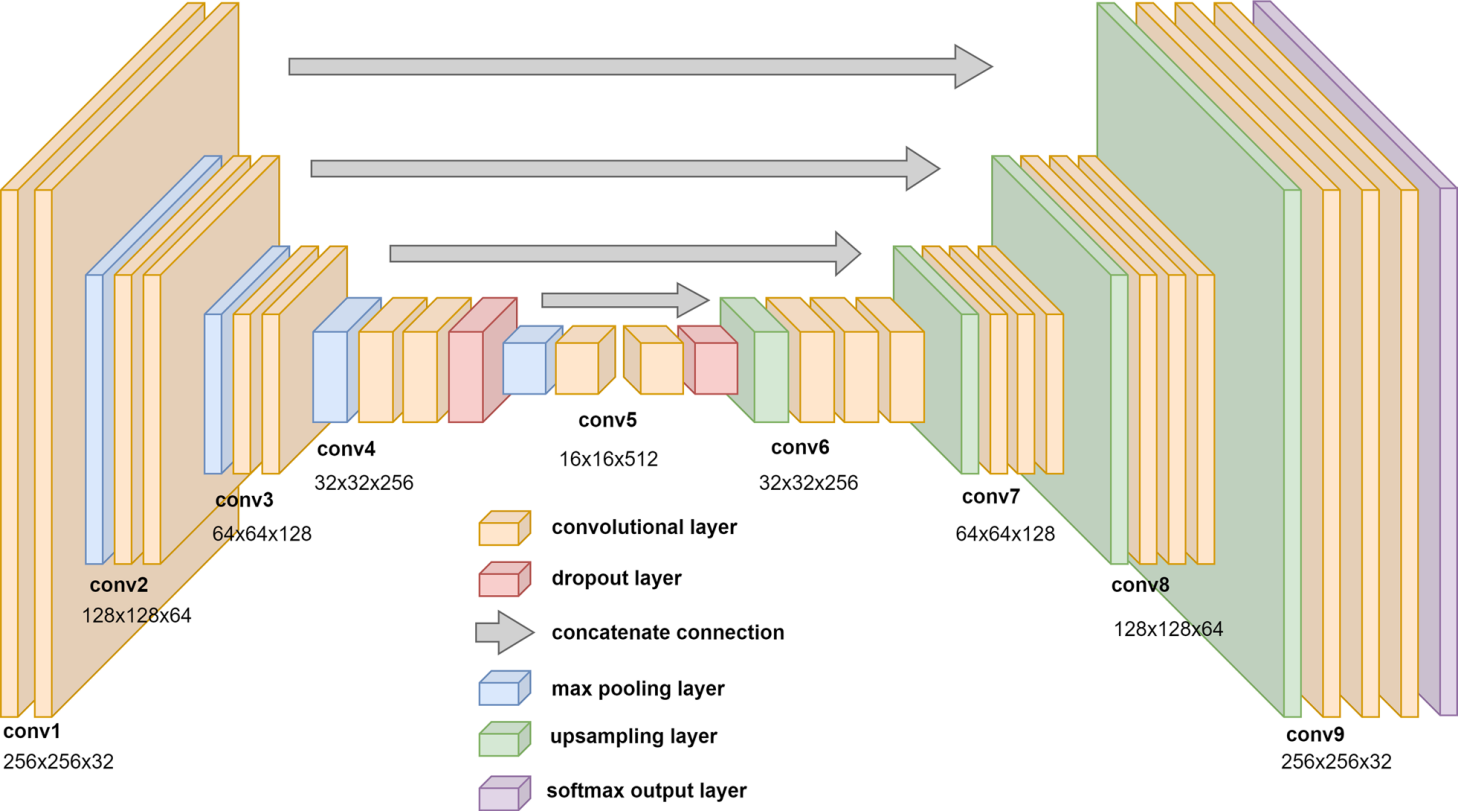
U-Net convolutional neural network architecture

The CNN was modeled after the well-established U-Net structure [17]. Adaptations of the U-Net structure have proven successful in a number of medical contexts, from cell analysis [18, 19] to segmentation tasks [11] and beyond [20]. Figure 2 shows a visual of the CNN architecture. The CNN was built and trained in Python 3.7, using the deep learning packages Keras and TensorFlow, versions 2.3.1 and 2.2.0, respectively. Each OAR was trained in a separate training iteration using the same model architecture. The model used the Adam optimizer with an initial learning rate of 5 × 10^−5^ [21]. A 0.25-factor learning rate reduction was applied in the case of a training plateau, down to a minimum learning rate of 1 × 10^−6^. The loss function, L, was defined as the sum of the Dice loss and the binary cross-entropy loss.

1 $$\begin{array}{*{20}c} {L = \left( {1 - \frac{{2\left| {Y \cap \hat{P}} \right|}}{{\left| Y \right| + \left| {\hat{P}} \right|}}} \right) + \left( { - \left[ {Y\log \hat{P} + \left( {1 - Y} \right)\log \left( {1 - \hat{P}} \right)} \right]} \right)} \\ \end{array}$$

In Eq. (1), $Y$ represents the ground truth array and $\hat{P}$ represents the predictions generated by the CNN. The model was trained for 200 epochs with a batch size of 32. The last 15% of each training set was separated as validation data, which was used to benchmark the training at the end of each training epoch.

On-the-fly data augmentation was built into the training process using the tools in Keras to randomly modify training images as they are passed to the model. This, along with the dropout layers, prevents the model from overfitting to training data. These modifications were limited to maximums of 10° rotation, 15% translation (vertically or horizontally), 10% scaling, or 0.2° shear. Once the augmentation was added, independent training cycles were performed on each of the 11 OARs.

Once all training iterations were completed, a predicted structure set containing all 11 OARs was generated for each of the 19 test patients for evaluation. A threshold of 0.33 was applied to binarize the pixel values. Only the largest single volume was accepted as the CNN’s prediction, and any discontinuous positive pixels were discarded as outliers. Additionally, axial limits were applied according to the means and standard deviations of organ shape training data to prevent overprediction on the following OARs: parotid glands, submandibular glands, brainstem, and larynx. The mean height of each organ, plus either one or two standard deviations, depending on the organ, was set as a maximum height boundary for the prediction, and prediction data in excess of this limit were discarded.

### Comparison to atlas method

The workflow converts the post-processed deep learning contours (DLCs) into a DICOM-compliant structure set file for direct comparison to the ground truth manual contours. Similarly, DICOM structure set files were generated on the 19 test patients using an atlas-based method available in the MIM6 application (MIM version 6.9.4; MIM Software Inc., Beachwood, OH) as tuned for our clinic. The atlas-based contours (ABAS) were used as a performance benchmark to ensure that our developed CNN workflow did not generate inferior contours. To compare these two methods, each method was scored against the accepted ground truth contours using three quantitative performance metrics: Dice similarity coefficient (DSC), mean surface distance (MSD), and 95^th^ percentile Hausdorff distance (HD). A paired, two-tailed Student *t* test was conducted to assess the statistical significance of performance differences. In this analysis, the null hypothesis was defined as no performance difference between the DLC and ABAS. A standard *p* value threshold of 0.05 was used. A *p* value below this threshold indicated a statistically significant difference in the two results sets, allowing the null hypothesis to be rejected [22].

To properly contextualize the performance of each method in terms of potential benefit to the clinical workflow, both sets of contours were evaluated by a trained physician for acceptability. For each of the 19 test cases, each contour was scored between 1 and 5 according to the following scale: 1, no changes necessary; 2, mild changes (not clinically significant); 3, moderate changes (clinically significant); 4, unacceptable contour, discard and segment manually; 5, structure failed to contour [23]. Although atlas-based workflows are available in many clinics, the output requires manual review before use in treatment planning [24, 25]. Thus, a valuable additional metric is the required correction time. For our test set, the evaluating physician recorded the time required for any corrections that were necessary to bring the contour to clinical acceptability. Only time spent actively editing the contour was recorded.

### Framework evaluation

The framework’s ability to generate DICOM-compliant structure set files—an essential prerequisite to enable physician evaluation in DICOM viewing software—was evaluated by using it to generate a file for each of the 19 test cases. The process of generating these structure sets allowed for two important evaluations. First, it measures the expected performance of the framework against our previously stated usability criteria. Second, it allows an estimation of the expected time required to run the process—an important factor for clinical implementation. For this, the only time assessed was that required after the DICOM image files were successfully transferred to temporary storage on the server, because the file transfer speed is a function of technology infrastructure and is independent of our framework. Processing time for the deep learning framework was assessed on a custom computer with an Intel i7-9700K processor, 32 GB of RAM, and an Nvidia GeForce RTX 2060 Super GPU.

## Results

**Fig. 3:**
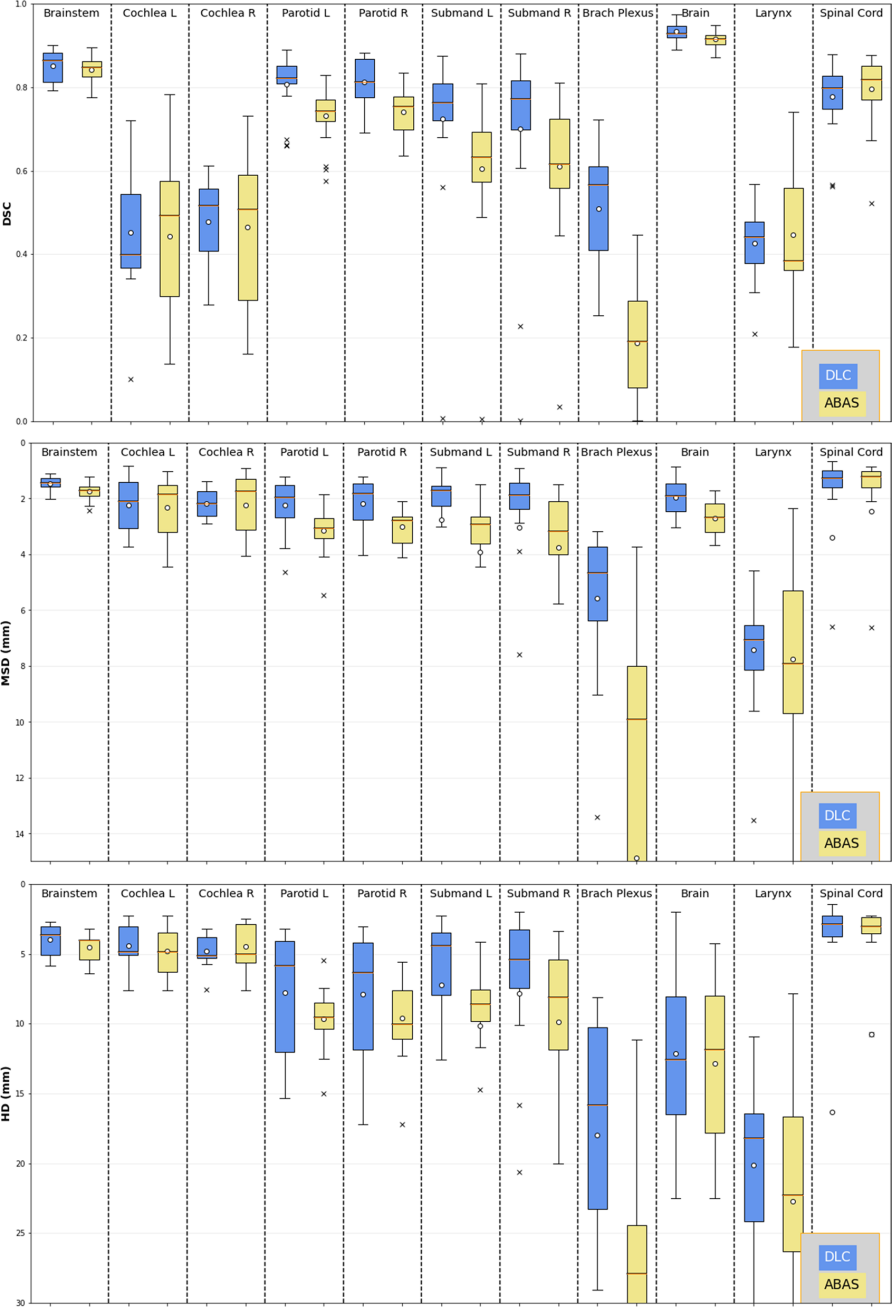
Box-and-whisker plots of each method, for each OAR, for all three quantitative metrics. For each plot, the *y* axis is oriented such that preferable metrics scores are at higher positions on the plot

Box-and-whisker plots provide a useful visualization of the distribution of results with more detail than the standard deviation alone. The independent box-and-whisker plots for all OARs considered can be seen for DSC, MSD, and HD in Fig. 3, and the mean and standard deviation of each OAR is shown in Table 2. For a more in-depth examination of the results, the per-patient scores on each metric are provided in the additional files [see Additional file 1]. These results and statistical evaluation provide a comprehensive view of the performance of each method on each OAR. For DSC, higher scores are preferable: the scores for DLCs were significantly better than for ABAS for the submandibular glands as well as for the brainstem, brain, and brachial plexus (Fig. 3). For the MSD, lower values indicate closer alignment of the contours with the ground truth: similar to that for DSC, DLCs performed better for the submandibular glands, brainstem, brain, and brachial plexus but performed comparably to ABAS for the other OARs. For the HD, lower values indicate reduced maximum error: the DLCs were significantly better than ABAS for the submandibular glands, brachial plexus, and brainstem.

**Table 2:** Means and standard deviations for quantitative metrics on each method

ROI		Brainstem	Brain	Cochlea L	Cochlea R	Parotid L	Parotid R	Submand. L	Submand. R	Larynx	Spinal Cord	Brachial Pl
DSC	DLC	**0.852 ± 0.037**	**0.933 ± 0.021**	0.452 ± 0.149	0.477 ± 0.106	**0.808 ± 0.066**	**0.812 ± 0.057**	**0.724 ± 0.183**	**0.700 ± 0.217**	0.426 ± 0.089	0.777 ± 0.086	**0.509 ± 0.139**
	ABAS	0.842 ± 0.033	0.916 ± 0.018	0.442 ± 0.180	0.466 ± 0.184	0.731 ± 0.069	0.741 ± 0.059	0.605 ± 0.160	0.610 ± 0.173	0.447 ± 0.146	0.797 ± 0.084	0.186 ± 0.130
MSD	DLC	**1.466 ± 0.260**	**1.955 ± 0.668**	2.237 ± 0.872	2.185 ± 0.469	**2.257 ± 0.951**	**2.185 ± 0.883**	**2.755 ± 3.891**	3.055 ± 3.893	7.441 ± 1.917	3.394 ± 5.535	**5.587 ± 2.553**
	ABAS	1.760 ± 0.309	2.702 ± 0.594	2.329 ± 1.046	2.234 ± 1.017	3.153 ± 0.731	3.012 ± 0.636	3.914 ± 3.985	3.756 ± 3.111	7.755 ± 3.264	2.468 ± 4.002	14.88 ± 11.51
HD	DLC	**3.952 ± 1.018**	12.13 ± 5.968	4.404 ± 1.477	4.780 ± 1.081	7.747 ± 4.331	7.881 ± 4.521	**7.231 ± 7.483**	**7.840 ± 7.511**	20.12 ± 5.163	16.35 ± 32.89	**18.45 ± 9.956**
	ABAS	4.526 ± 1.032	12.82 ± 5.061	4.767 ± 1.678	4.474 ± 1.562	9.656 ± 2.029	9.586 ± 2.678	10.14 ± 7.626	9.844 ± 6.965	22.74 ± 8.916	10.76 ± 23.09	37.26 ± 22.65

Cells with bold text indicate statistically significant performance difference (p-value > 0.05, paired two-tailed Student t-test)

DSC: Dice Similarity Coefficient. MSD: Mean Surface Distance (reported in mm). HD: 95th Hausdorff Distance (reported in mm). DLC: Deep Learning Contours; ABAS: Atlas-based Contours. Submand: Submandibular gland. Brachial Pl: Brachial plexus

In Table 2, cells with bolded values indicate that a given method scored statistically better than the other method for that OAR. Notably, there was no OAR/metric combination for which the atlas method achieved significantly better performance. For four of the OARs, neither method was statistically better across any of the three metrics. However, for the other seven OARs, the DLC was significantly better according to at least two of the three metrics.

**Fig. 4:**
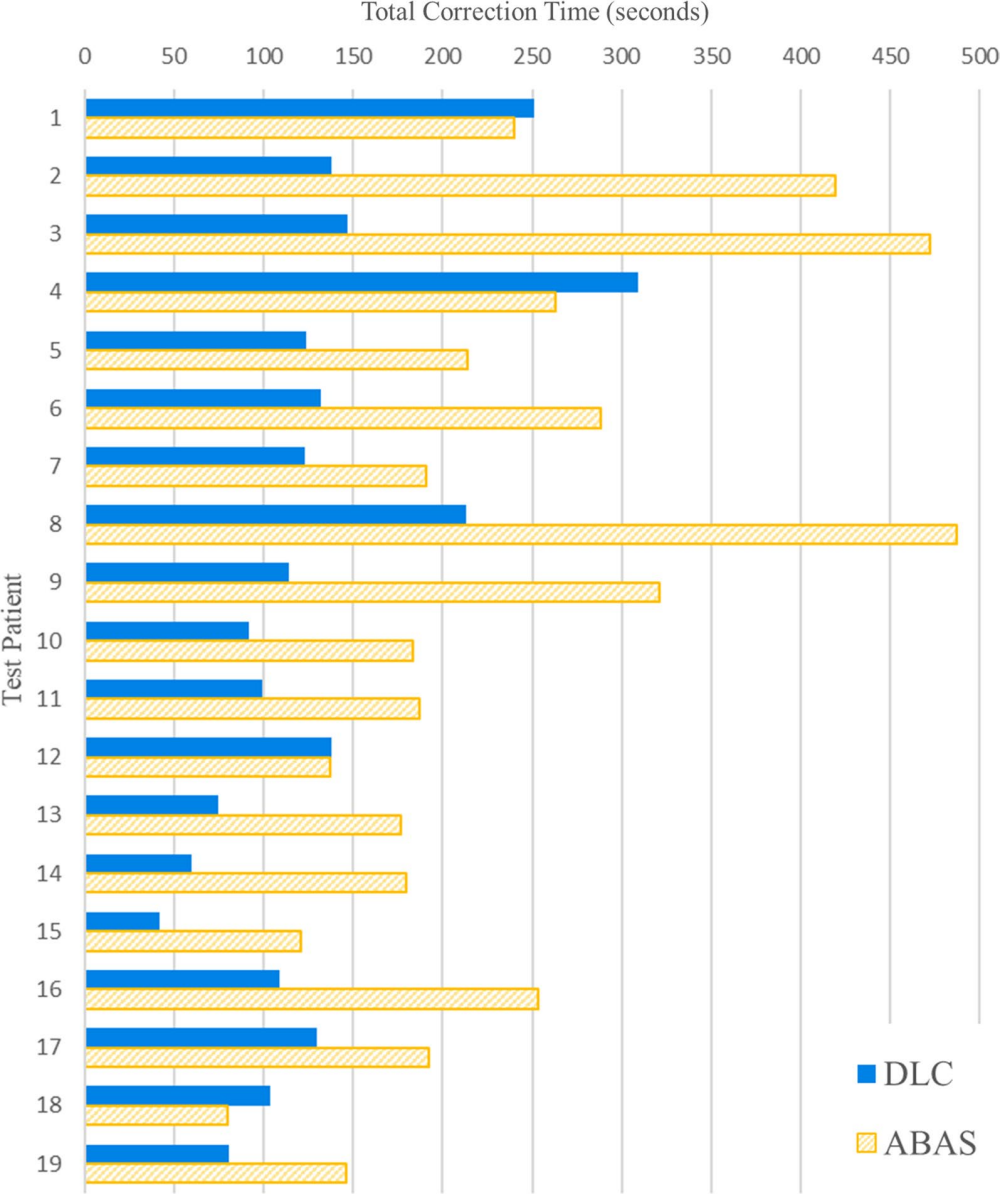
Bar plot of total correction time required in physician review per test patient

Statistical significance is a useful initial review, but physician acceptance is the most meaningful criterion for a tool being considered for clinical application. Table 3 shows the results of the physicians’ qualitative scoring. Although the DLCs often required minor adjustments, the ABAS more frequently required substantial correction. Accordingly, DLCs required less overall correction time than ABAS for 17 of 19 test patients, as seen in Fig. 4.

**Table 3:** Qualitative scoring by MD review for each OAR of each autocontouring method

ROI	DLC SCORING				
	1	2	3	4	5
Brain	74%	26%	–	–	–
Brainstem	89%	11%	–	–	–
Cochlea L	100%	–	–	–	–
Cochlea R	95%	5%	–	–	–
Parotid L	5%	95%	–	–	–
Parotid R	5%	95%	–	–	–
Submandibular L	74%	21%	5%	–	–
Submandibular R	79%	16%	5%	–	–
Brachial plexus	32%	53%	15%	–	–
Spinal cord	69%	31%	–	–	–
Larynx	–	11%	89%	–	–

ROI	ABAS SCORING				
	1	2	3	4	5
Brain	100%	–	–	–	–
Brainstem	100%	–	–	–	–
Cochlea L	63%	37%	–	–	–
Cochlea R	69%	31%	–	–	–
Parotid L	21%	79%	–	–	–
Parotid R	16%	79%	5%	–	–
Submandibular L	5%	58%	37%	–	–
Submandibular R	15%	53%	21%	11%	–
Brachial plexus	26%	16%	11%	47%	–
Spinal cord	100%	–	–	–	–
Larynx	–	26%	74%	–	–

DLC: Deep Learning Contour; ABAS: Atlas-based Contours. Grading scale: 1—No changes necessary; 2—Mild changes (not clinically significant); 3—Moderate changes (clinically significant); 4—Unacceptable contour, discard and segment manually; 5—Structure failed to contour

**Fig. 5:**
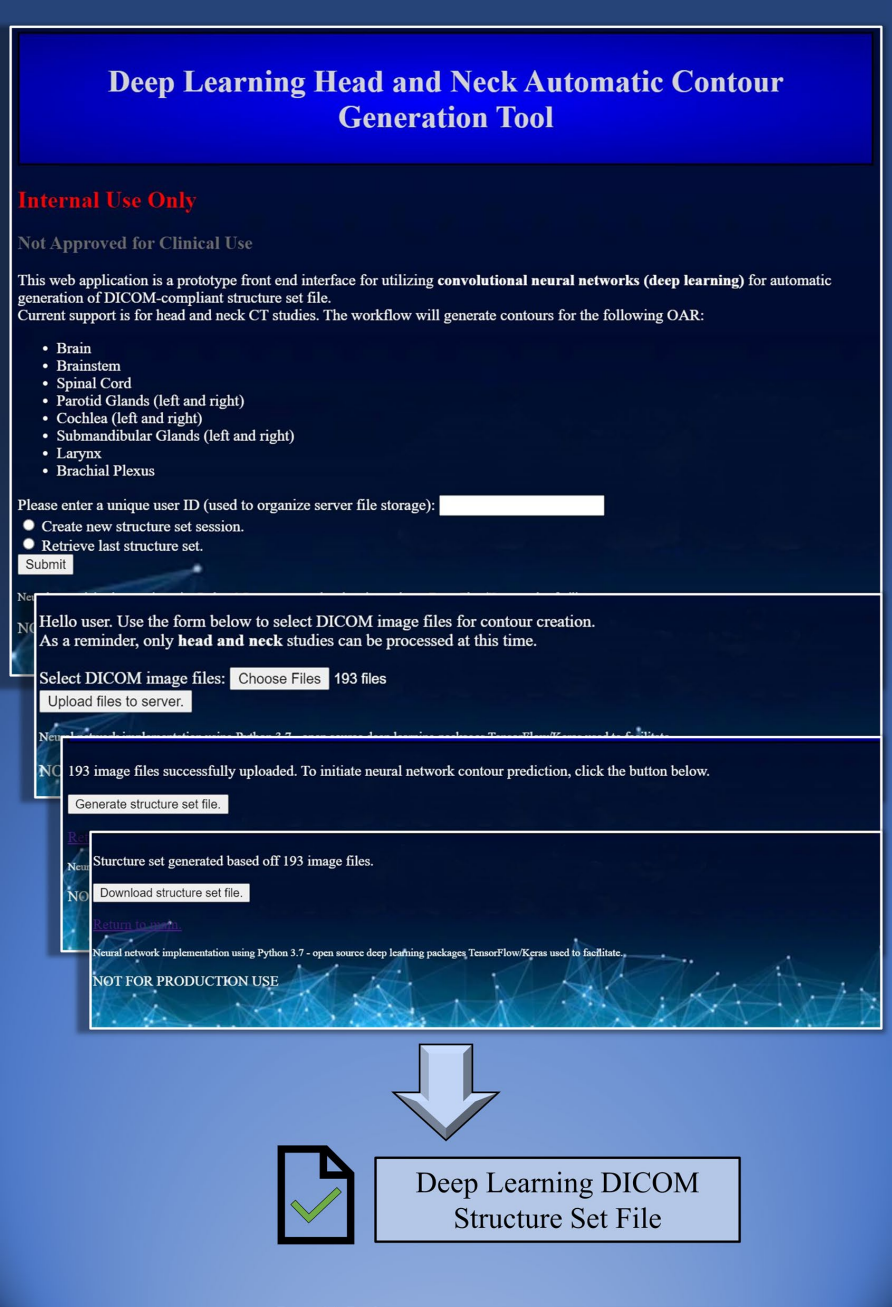
Example of provided browser interface sequence for simple deep learning contour generation

The generation of the test patient structure sets validated the first framework usability criterion—receiving DICOM image files as input and returning a DICOM structure set file as output—because the process was performed as it would be in a clinical setting. The second usability criterion—the capacity to retrain the network on new data—is fundamentally built into the framework, because the initial training was an essential task in the development of the framework. The final usability criterion—a browser-based, no-code interface for use—was also used in the generation of the test patient structure sets. Images of this interface can be seen in Fig. 5.

## Discussion

The results of the quantitative and qualitative evaluations support the acceptability of the CNN framework for generating initial contours of the 11 OARs considered. Although the focus of this work was making a deep learning framework accessible, it is essential to establish the competence of the model. Figure 3 shows the preferable performance of the DLC to the ABAS on nearly all OARs. The difference is clearest on the glandular OARs and brachial plexus, but improvement can also be seen on the brainstem and the brain. The larynx, spinal cord, and cochleae are less clear, and the scores for the two methods are much closer. From the results of the statistical analysis, we know that there is not a statistically significant difference in performance for every OAR, even when the box-and-whisker plots show separation. A *p* value of greater than 0.05 for a Student *t* test does not mean that the two methods perform equally well, only that neither method emerged as statistically superior. However, the fact that the ABAS was not statistically significantly superior for any OAR, combined with the favorable qualitative review by the physician, gives us confidence that using the DLC in place of the ABAS in the clinical workflow would not result in decreased contour quality. The atlas-based method generated contours approximately 30 s faster; this is likely attributable to the server-based computation resources used for the atlas workflow, whereas the deep learning framework was run on an individual computer. Time–cost optimization of the deep learning pipeline would be a useful future expansion of this work. Overall, we expect that the DLC will slightly increase contour quality while decreasing the time required for correction.

The performance of our DLC framework is comparable to the results published in similar works. The work of van Dijk et al. is the most similar to ours: they used an ensemble of two-dimensional CNNs to produce head and neck contours and compared the results to an atlas-based method both qualitatively and quantitatively [6]. They reported better performance with DLC for glandular OARs, with a DSC of 0.81 ± 0.08 compared to 0.72 ± 0.10 for ABAS, which closely matches our results for the parotid glands. Similar quantitative performance was noted for other OARs common to both studies, such as brainstem and spinal cord. However, the work by van Dijk et al. [6] included commercial deep learning software, which is needed to reproduce their study. Zhong et al. recently reported a similar U-Net approach with qualitative and quantitative evaluation [12]. They used a single multi-class CNN and described a train-retrain cycle. The performance of this network on the first pass is similar to or better than ours for shared OARs, with DSC values of 0.79 for parotid glands, 0.79 for brainstem and spinal cord, and 0.72 for larynx; these scores improved further after their retrain process. Apart from a brief description of the neural network structure they used, there is little information about data processing or implementation and no code is provided [6]; thus, their work is also challenging to reproduce. Nikolov et al. published the results of a three-dimensional CNN that performed slightly better than our framework, but the code for their method “makes use of proprietary elements” and therefore cannot be shared [26]. Once again, reproduction is not possible. The output performance of our framework is in line with other published work, but we consistently found that missing or proprietary information prevented reasonable reproduction or implementation of the methods.

We acknowledge that training and validating our framework on internal data sourced from our clinic does not demonstrate the generalizability of the pre-trained CNN offered as part of the framework. If the CNN learned image features unique to our imaging protocols, it may perform worse on images from other sources. This is why the ability to retrain the CNN is crucial to the overall framework. Rather than attempt to create a CNN that universally contours CT data, we offer the scripts for simple retraining and performance evaluation. This allows researchers to adapt our methods specifically to their environments using their own image data.

Apart from the neural network itself, an important benefit of our framework is the ability to interface directly with DICOM files as input and output. Typically, a researcher with interest in deep learning needs to reproduce a substantial amount of code to extract data and prepare it for input to a CNN training process. Additionally, scripts to process the output of the CNN into a clean, compliant DICOM structure set file are essential. All of these scripts are contained in our framework and support minimal-configuration use. After an initial setup, even users who do not know programming can generate contours. By providing these tools as part of our framework, we offer an advanced starting point for entry into deep learning research.

Although we view this framework as primarily a research tool, it could be expanded and inserted into a regular clinical workflow. The pre-trained OARs in the framework were not comprehensive, but the provided training tool allows clinics to customize the CNN training to their data and needs. By using an internal computer to act as a server, a single configuration of the framework would be available to all clinical personnel through their web browsers without requiring the end users to interface with Python. Alternatively, some vendors of treatment planning software offer scripting application programming interfaces that can integrate customized capabilities into the treatment planning workflow [27–29]. Work is underway at our clinic to incorporate a more robust framework, based in concept on the work presented here, to enable clinicians with various levels of technical expertise to access deep learning research tools.

## Conclusions

In this work, we present a feasible solution to bridge the gap between cutting-edge deep learning research and everyday clinical practice. With a small setup effort, our framework provides free, no-code utilization of deep learning autocontouring. This framework is deliberately designed to facilitate access to deep learning research: it includes all scripts and tools necessary to explore deep learning contouring research using DICOM files. We view this as an important contribution toward the treatment planning workflow. As the field continues to advance and deep learning techniques become more ubiquitous, familiarity with the techniques will be increasingly important for clinical personnel, and the need for researchers to create approachable point-of-entry tools for clinics that are not bound by commercial contracts will continue to grow.

## Supplementary Information

**Additional file 1.** Quantitative metric results and statistical analysis. This file contains per-test case result scores from each of the 19 test patients for each considered OAR for both DLC and ABAS. These data formed the basis of Table 2, which reports the means, standard deviations, and statistical significance of results per the Student *t* test.

## Data Availability

Data used to train and validate the neural network were internally sourced and are available upon reasonable request to the corresponding author contingent on Roswell Park Comprehensive Cancer Center approval. All code used for development and deployment of this research is available per the following information: Project name: Deep Learning Autosegmentation Pipeline. Project home page: https://github.com/jasbach/HN_UNet_Autosegmentation_Tool. Operating system(s): Platform independent. Programming language: Python. Other requirements: Python 3.6 or higher, and the following Python packages: Tensorflow 2.3.0, Keras 2.4.3, NumPy 1.19.2, Pydicom 2.1.2, Opencv-python 4.5.1.48, Flask 1.1.2 (if utilizing browser-based interface). License: MIT License. Any restrictions to use by non-academics: No restrictions.

## Abbreviations

CNN: Convolutional neural network
OAR: Organ-at-risk
DLC: Deep learning contours
ABAS: Atlas-based contours
DSC: Dice similarity coefficient
MSD: Mean surface distance
HD: 95Th percentile Hausdorff distance
